# Multiplexed Weak Waist-Enlarged Fiber Taper Curvature Sensor and Its Rapid Inline Fabrication

**DOI:** 10.3390/s21206782

**Published:** 2021-10-13

**Authors:** Duo Yi, Lina Wang, Youfu Geng, Yu Du, Xuejin Li, Xueming Hong

**Affiliations:** 1 College of Physics and Optoelectronic Engineering, Shenzhen University, Shenzhen 518061, China; yiduo@szu.edu.cn (D.Y.); wanglina2017@email.szu.edu.cn (L.W.); duyu@szu.edu.cn (Y.D.); xmhong@szu.edu.cn (X.H.); 2 School of Science and Engineering, Chinese University of Hong Kong, Shenzhen 518172, China; lixuejin@szu.edu.cn

**Keywords:** curvature sensor, fiber interferometer, waist-enlarged fiber taper

## Abstract

This study proposes a multiplexed weak waist-enlarged fiber taper (WWFT) curvature sensor and its rapid fabrication method. Compared with other types of fiber taper, the proposed WWFT has no difference in appearance with the single mode fiber and has ultralow insertion loss. The fabrication of WWFT also does not need the repeated cleaving and splicing process, and thereby could be rapidly embedded into the inline sensing fiber without splicing point, which greatly enhances the sensor solidity. Owing to the ultralow insertion loss (as low as 0.15 dB), the WWFT-based interferometer is further used for multiplexed curvature sensing. The results show that the different curvatures can be individually detected by the multiplexed interferometers. Furthermore, it also shows that diverse responses for the curvature changes exist in two orthogonal directions, and the corresponding sensitivities are determined to be 79.1°/m^−1^ and –48.0°/m^−1^ respectively. This feature can be potentially applied for vector curvature sensing.

## 1. Introduction

Curvature sensing is essential in various fields such as smart robot [1], engineering structure [2] and medical image [3], etc. When compared with the traditional resistive curvature sensors, the optical fiber curvature sensor owns the advantages such as flexibility, small size, low cost, immunity to electromagnetic interference and easiness to form sensing network [4,5]. Nowadays, optical fiber curvature sensors based on intermodal interferometer have been widely reported [6,7,8]. Particularly in recent years, the use of the microstructured fiber as the transducing platform promotes the exploitation of various curvature sensors based on photonic crystal fiber [9], hollow-core fiber [7], few-mode fiber [10], four-core fiber [11] or Fabry-Perot [12], etc. However, for these intermodal interferometers, post-processing techniques such as fiber tapering [13], side-polishing [14] and cladding etching [15] are always used to modify the fiber structure and convert part of fundamental mode into the high-order mode in order to construct an optical arm. It inevitably results in a large insertion loss and structure fragileness, which makes the essentially multiplexed curvature sensing difficult to be achieved with these techniques.

In our previous work [16], a waist-enlarged fiber taper sensor has been proposed for high-temperature sensing. It has a remarkable and distinguishable fiber structure, a strongly waist-enlarged taper, to excite high-order fiber modes. This type of fiber taper can be named as a strong waist-enlarged fiber taper (SWFT) and has been comprehensively used to construct fiber sensors for the sensing applications such as temperature [16], strain [17], refractive index [18,19] and humidity [20]. It owns the advantages of robust structure, easy fabrication and flexible mode excitation, etc. However, it needs repeated cleaving and splicing during the fabrication process, and the structural health of the sensor still needs to be improved due to the embedded splicing points in the fiber. Besides, similar to the intermodal interferometers mentioned in the previous paragraph, the fabricated SWFT also faces the problem of large transmission loss, which results in a poor signal-noise ratio, but in contrast, presents a moderate spectral dynamic range over 10 dB [16].

In this study, a weak waist-enlarged fiber taper (WWFT) and its rapid inline fabrication method are further proposed, and the application for multiplexed curvature measurement is studied. The WWFT could be fabricated rapidly with a weak electric arc discharge released on the inline sensing fiber, and the repeated cleaving-splicing process is no longer needed. It has no fusion points and no obvious structural deformation in appearance compared with general single mode fiber (SMF). Therefore, the insertion loss of the WWFT-based interferometer is greatly reduced, which makes it possible to realize multiplex curvature sensing with phase-demodulation scheme. Unfortunately, the WWFT device presents a relatively low spectral dynamic range. Finally, the experimental results of curvature measurement demonstrate that the proposed sensor has diverse responses for the curvature changes in two orthogonal directions, showing its great potential applications in vector curvature sensing.

## 2. Sensor Fabrication

**Figure 1:**
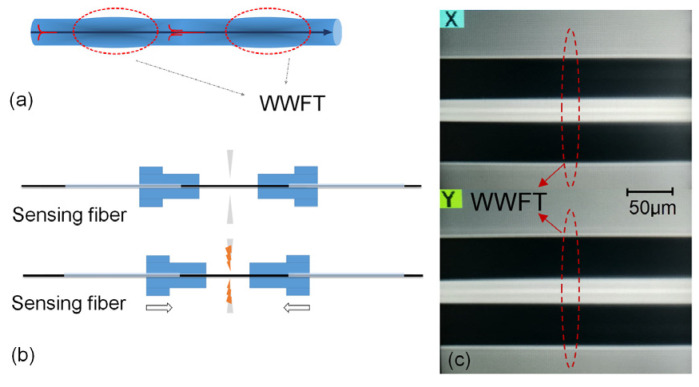
Schematic of WWFT and its fabrication: (**a**) Schematic of WWFT and the constructed intermodal interferometer; (**b**) Inline fabrication of WWFT; (**c**) XY side views of the fiber taper with overlap of 20 μm. The red circle represents the fiber taper location.

Figure 1a shows the schematic of WWFT and the constructed intermodal interferometer, which has no difference in appearance with the SMF. Figure 1b shows the specific inline fabrication process of WWFT. It is fabricated by using the manual splicing mode of the fiber splicer (Fujikura Ltd., FSM 60S, Tokyo, Japan) in our experiment. Firstly, two segments of SMFs with polymer coating stripped and end cleaved are prepared, and they are separately put onto the motor stages of the fiber holder. The motors are impelled and the fiber end faces are aligned, then the splicing process is temporarily suspended. In this step, the motors are adjusted to avoid fiber bending during arc discharge. Secondly, an SMF whose polymer coating has been stripped is used to replace the aligned fibers on the motor stages, and arc discharge is then released when the two motor stages are pushed forward with a designed overlap distance, defined as *L*_vp_. In this study, three *L*_vp_ values of 10, 20 and 30 μm are selected to fabricate the WWFT in the experiment for comparison. Using this method, the WWFT is rapidly embedded into the sensing SMF fiber. The arc discharge intensity and duration time are set as ‘standard mode’ and 200 ms respectively. Note that, if the motor can be automatically aligned, the aligned process with two cleaved fibers in the first step is no longer needed and the duration of fabrication process could be shortened to just a few seconds. Therefore, the WWFT is possible to be massively fabricated by a specially designed arc releasing electrical and mechanical structure for rapidly arranging a large-scale sensing network. Figure 1c shows the side views of the WWFT. It can be seen that, different from the SWFT, it has no difference in appearance with SMF, and no splicing point is embedded in the sensing fiber, which gives a strong physical structure and ultralow insertion.

**Figure 2:**
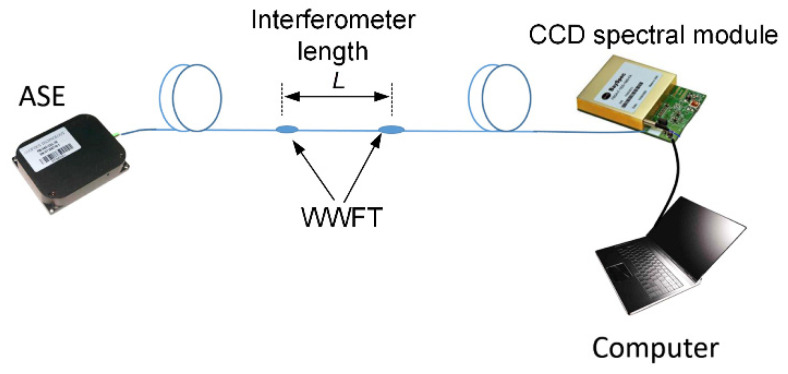
Experiment setup of WWFT-based interferometer. ASE: amplified spontaneous emission, CCD: charge-coupled device, WWFT: weak waist-enlarged fiber taper.

Next, two WWFTs are sequentially embedded into the sensing SMF to construct an intermodal interferometer, as shown in Figure 2. For the experimental test, the WWFT-based interferometer is connected with ASE light source (Golight Tech., ASE-C&L-10, Shenzhen, China) and CCD spectral module (Bayspec Inc., FBGA-1525-1610, Fremont, CA, America) for transmission spectrum acquisitions. The acquired spectra are then analyzed by using a LabVIEW program for Fast Fourier Transform (FFT), and the peak amplitudes/phases of the spectra after FFT are individually discussed. The schematic diagram of the experimental setup is shown in Figure 2.

## 3. Spectral Characterization of WWFT-Based Interferometer

**Figure 3:**
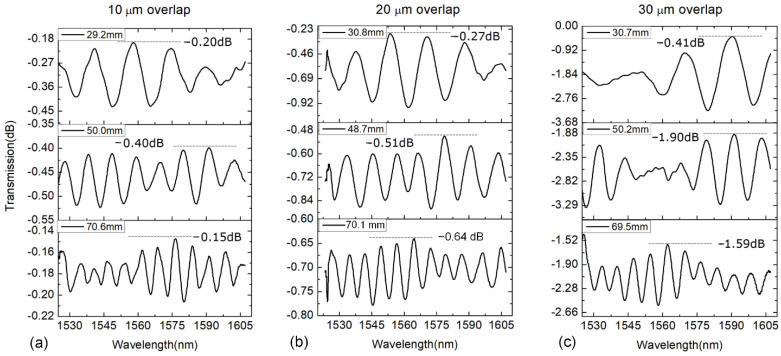
Transmission spectra of WWFT-based interferometers with different overlap values of (**a**) 10 μm, (**b**) 20 μm and (**c**) 30 μm, respectively.

For the following analyses, sensors with different overlap values *L*_vp_ (10, 20 and 30 μm) are experimentally fabricated for comparison. Besides, various MZ interferometer samples with different interferometer lengths of *L* are also prepared. Figure 3 shows the transmission spectra of the WWFT-based interferometers with overlaps of 10 μm, 20 μm and 30 μm, respectively. We can observe that the induced insertion losses could be as low as 0.15 dB when *L*_vp_ = 20 μm. It increases slightly with the overlap distance enlarged since more high-order modes are excited and experience large transmission loss. From the figure, it also can be seen that if the overlap distance remains the same, the fringe contrast decreases as the interferometer length elongates. For example, when *L*_vp_ = 20 μm, the maximum fringe contrasts are 0.68 dB, 0.37 dB, 0.12 dB, respectively, for the interferometer lengths of 30.8 mm, 48.7 mm and 70.1 mm. Actually, when the interferometer length increases, the optical power ratio between sensing arm and reference arm of interferometer gradually decreases since the high-order mode suffers a higher transmission loss. Finally, it results in the deterioration of fringe contrast.

**Figure 4:**
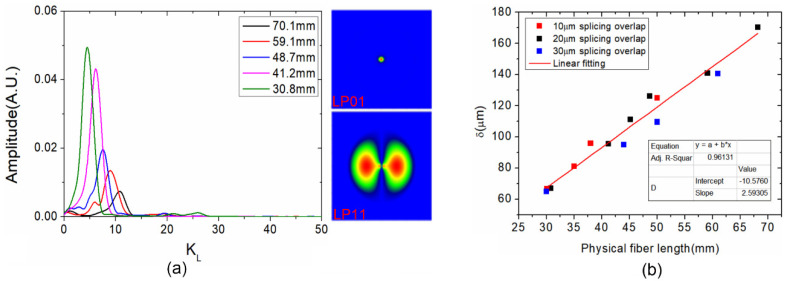
(**a**) Spectra after FFT for the WWFT-based interferometers with different interferometer lengths, inset: Electric field distributions of LP_01_ and LP_11_ mode; (**b**) Optical path difference versus interferometer length with overlaps of 10 μm, 20 μm and 30 μm, respectively.

Since WWFT is with non-adiabatic characteristics, part of the energy of LP_01_ is leaked and coupled into the high-order modes [21]. Next, in order to evaluate the specific mode order excited by WWFT, the Fast Fourier Transform (FFT) is applied to the transmission spectra in Figure 3. Herein, Figure 3b is selected, and another two sensors with interferometer lengths of 41.2 mm and 59.1 mm are supplemented for the following analyses. The results are shown in Figure 4a. For each interferometer with a certain physical length *L*, the optical path difference (OPD) *δ* could be calculated through the relationship of $\delta =cK_{Lm}/2\Delta \nu$ [22], where *K_Lm_* is the index of peak amplitude location in the FFT spectra, and ∆ν is the whole frequency range of CCD spectral module. It should be noted that *K_lm_* is a parameter with no dimension, and it varies with the frequency range of CCD spectral module. Hence, the calculated OPD itself is independent with the CCD module. Figure 4b illustrates the calculated OPD versus interferometer length *L* of the WWFT-based interferometer with different overlap distances of 10, 20 and 30 μm. It is well known that the optical path difference could be also expressed as δ=ΔnL, where Δ*n* is the difference of effective refractive index between LP_01_ and the excited high-order mode. Therefore, associating with the two different expressions of δ above, the Δ*n* could be determined in experiment, and the value is 2.59 × 10^−3^, which is very close to the theoretical value of 2.73 × 10^−3^ between LP_01_ and LP_11_ mode using beam propagation method. Hence we can infer that the excited high-order mode is LP_11_ mode. The electric field distributions of LP_01_ and LP_11_ mode are displayed as the inset of Figure 4a.

## 4. Results and Analyses

**Figure 5:**
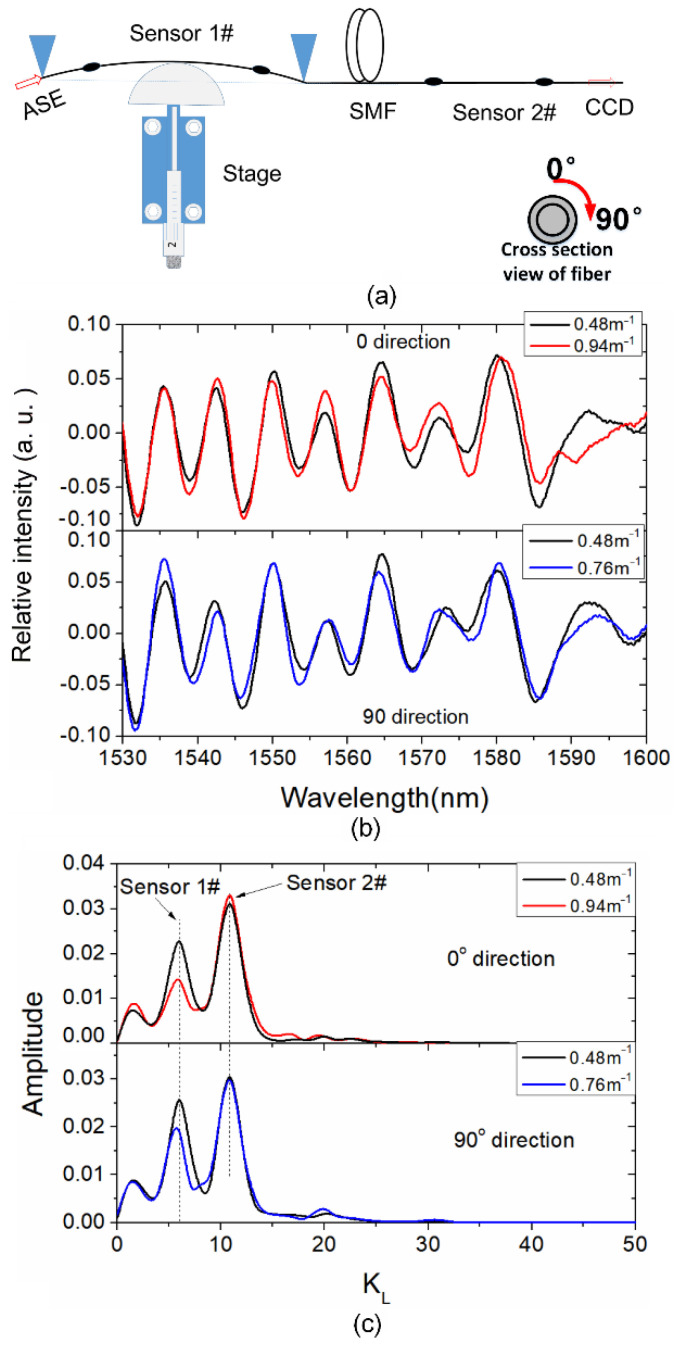
(**a**) Schematic of curvature test for the two cascaded WWFT-based interferometers; (**b**) Spectra and (**c**) Spectra after FFT for the WWFT-based interferometer 1# and 2# at 0° direction and 90° direction, respectively.

Owing to the low insertion loss of WWFT, the proposed sensor can be applied for curvature multiplex sensing. Figure 5a shows the schematic of the curvature measurement for two cascaded WWFT-based interferometers. The fiber sensor 1# and 2# are with interference lengths *L* = 41.7 mm and 69.5 mm, respectively. Besides, the overlap distances are set as 20 μm and 30 μm, respectively, for the two sensors. For the two cascaded interferometers, the total insertion loss is measured as approximately 2.5 dB, and it can be further reduced with a smaller overlap value. Besides, the amount of multiplexed sensors depends on the measurable spectral range. According to the equation $\delta =cK_{L}/2\Delta \nu$ in the last paragraph, a larger spectral range results in a shorter optical path difference, and further, a higher resolution in the spectra after FFT, hence more sensors can be multiplexed. During the test, sensor 1# is placed between two supporting points with a separated distance of 115 mm, while sensor 2# keeps still. A one-dimensional translation stage with circular metal head is placed at the center of the sensor 1#. With the micrometer screw thread pushed forward, a fiber arc shape is formed. The curvature can be calculated by the expression of ρ = 1/*R* = *8d*/(4*d*^2^ + *D*^2^) [23], where *R* is the curvature radius of the fiber arc induced by pushing the one-dimension fiber translation stage, *D* is the distance of two supporting points, and *d* is the bending displacement. In our case, *d ≪ D*, therefore, the curvature is simplified as ρ = 1/*R* = *8d*/*D*^2^.

Based on the experimental setup in Figure 5a, different external curvatures are applied on the sensor 1#, and the corresponding transmission spectra at the two orthogonal directions (0° and 90°, schematic of fiber rotation angle is indicated in the inset of Figure 5a) are shown in Figure 5b. It should be noted that the axis Y in Figure 5b represents the relative intensity; it is obtained by normalizing the transmission spectrum amplitude with the input power, and then the gravity center of the spectral intensity shifts to zero to eliminate the DC component, hence both positive and negative values are shown. It can be seen that the wavelength shifts in Figure 5b are insignificant and irregular, which signifies that the curvature information cannot be directly demodulated. Figure 5c shows the corresponding spectra after FFT; it can be seen that the peak amplitudes of sensor 2# almost keep still, while the peak amplitudes of sensor 1# become lower as the curvature increases in both 0° and 90° directions. This phenomenon is reasonable since the LP_11_ mode is more sensitive to the bending effect, which leads to an enlarged transmission loss. Hence, the fringe contrast at the second WWFT point starts to deteriorate and further results in the reduction of peak amplitude.

**Figure 6:**
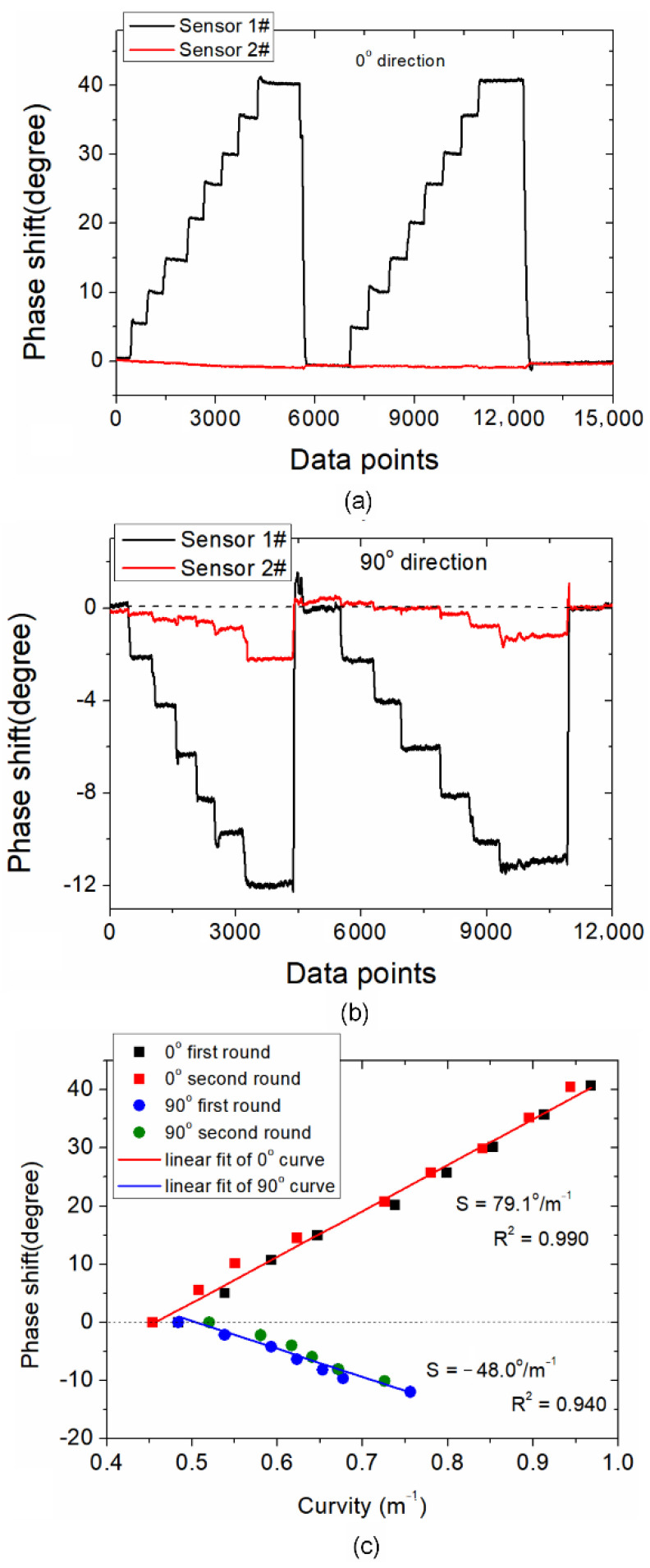
Phase responses of the WWFT-based interferometer 1# and sensor 2# in (**a**) 0° direction and (**b**) 90° direction; (**c**) Phase shift versus curvature in 0° and 90° direction.

Next, the continuous data acquisition is conducted, and the dynamic results are shown in Figure 6. The peak phases of spectra after FFT are continuously acquired as curvature varies between 0.48~0.94 m^−1^ (0° direction) °and 0.48~0.76 m^−1^ (90° direction). Figure 6a shows the phase shift when the WWFT-based fiber is placed at the initial position (defined as 0° direction). It can be seen that, when the curvature increases, the phase shift of sensor 1# is enlarged synchronously. Meanwhile, sensor 2# keeps almost unchanged, indicating that the curvature detections are independent between two sensors. Next, when the fiber sensor 1# is rotated to 90°, as shown in Figure 6b, we can see that the phase of sensor 1# shifts to the negative direction and presents different phase shifts when compared with those in 0° direction. This phenomenon signifies that the proposed sensor has diverse responses in two orthogonal directions, which can be further potentially applied for vector curvature sensing. Besides, for the 90° direction, we can also observe the cross-talk for sensor 2#. Theoretically speaking, no phase shift should occur for sensor 2# since no curvature is applied. This experimental phenomenon may be explained by the resolution ability of spectra after FFT, which is determined by the measurable spectral range of the CCD spectral module. As shown in Figure 5c, the peak amplitude of sensor 2# is easily affected by that of sensor 1# when the spectrum resolution ability is limited, and the cross-talk becomes more significant when the sensitivity decreases. Finally, the sensitivities in both 0° and 90° directions are shown in Figure 6c. The curvature sensitivities are determined to be 79.1°/m^−1^ and −48.0°/m^−1^, respectively. The different curvature sensitivities are mainly due to the anisotropic distribution of the excited high-order mode (LP_11_) in different axes. It should be explained that most of previous works relating to optical fiber curvature sensors are based on wavelength demodulation in unit of nanometer (nm) or amplitude demodulation in unit of decibel (dB). While in this study, the curvature sensing is realized based on phase demodulation in unit of degree (°). It should be noted that the wavelength and phase are two independent parameters which are inconvenient to convert to each other in our demodulation system. Hence, it is difficult to compare the curvature sensitivity performance presented in this study with the previously reported studies. Here, the sensitivity performance comparison is not discussed.

**Figure 7:**
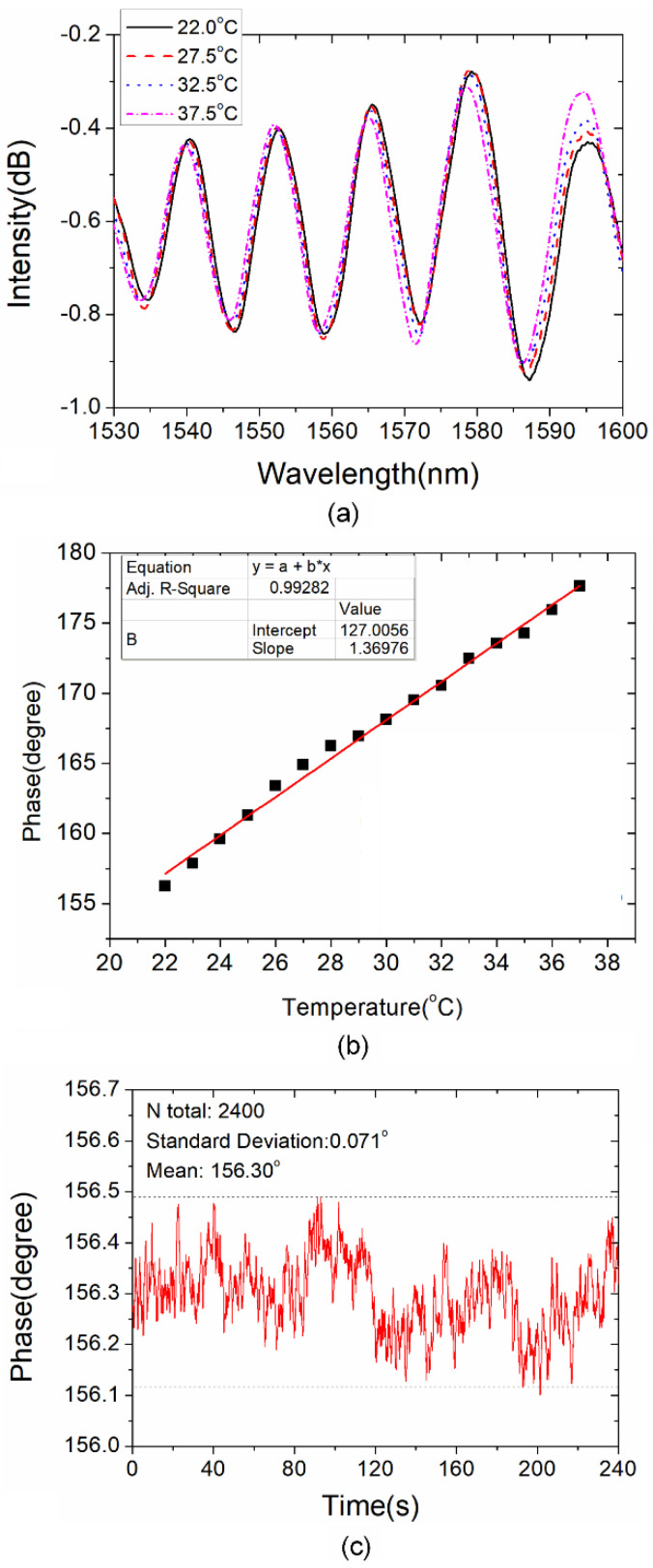
Temperature cross-sensitivity of WWFT-based interferometer: (**a**) Transmission spectra as temperature increases from 22 °C to 37.5 °C; (**b**) Phase shift versus temperature for the sensor with *L* = 41.2 mm and *L*_vp_ = 20 μm; (**c**) Continuous monitoring of phase fluctuation at 22.0 °C.

Finally, the temperature cross-sensitivity of the presented WWFT-based interferometer is discussed. The sensor is heated from 22.0 °C to 37.5 °C. Figure 7a shows the transmission spectra of the sensor with interferometer length of 41.2 mm and overlap value of 20 μm. We can hardly observe the wavelength shift with wavelength demodulation scheme. Then, based on phase demodulation scheme, the phase shift versus temperature is indicated in Figure 7b, and the temperature cross-sensitivity is determined to be 1.37 °/m^−1^. When compared with the curvature sensitivities mentioned above, the temperature cross-sensitivity is insignificant. Besides, the phase fluctuation is monitored for 4 min at 22 °C, and the result is shown in Figure 7c. The recorded standard deviation is determined to be 0.071°. Here, we use three times the standard deviation as the phase resolution, i.e., 0.213°. Hence, the curvature resolutions of the proposed sensor are determined to be around 0.213/79.1 = 2.69 × 10^−3^ m^−1^ at 0° direction and 0.213/48.0 = 4.44 × 10^−3^ m^−1^ at 90° direction.

## 5. Conclusions

This study demonstrates a multiplexed WWFT-based curvature sensor and its rapid inline fabrication. When compared with the other types of fiber taper, the proposed WWFT has no difference in appearance with the SMF, which greatly reduces the insertion loss. The WWFTs could be rapidly embedded into the inline sensing fiber without the repeated cleaving-splicing process and splicing point, which enhances the solidity of sensor. Owing to the low transmission loss which could be as low as 0.15 dB, the WWFT-based interferometer can be further multiplexed based on phase-demodulation scheme. The experimental results verified that curvature variation can be dynamically detected for two cascaded WWFT-based interferometers. Besides, the proposed sensor shows diverse responses for the curvature changes in two orthogonal directions, and the sensitivities are determined to be 79.1°/m^−1^ and −48.0°/m^−1^. Correspondingly, the curvature resolutions in two orthogonal directions are estimated as 2.69 × 10^−3^ m^−1^ and 4.44 × 10^−3^ m^−1^, respectively. This result confirms the feasibility for vector curvature sensing applications in the future.
